# Abnormal femoral trochlea morphology is a risk factor for secondary injury of anterior cruciate ligament after reconstruction

**DOI:** 10.1097/MD.0000000000036786

**Published:** 2024-01-05

**Authors:** Qiangqiang Cai, Dongqin Wang,, Liang Yan, Hailin Kuang, Wubing Tang, Zhihai Min, Xin Wang,

**Affiliations:** a The Third Hospital of Nanchang, Nanchang, Jiangxi, People’s Republic of China; b The First Affiliated Hospital of Nanchang University, Nanchang, Jiangxi, People’s Republic of China; c Hebei Medical University Third Hospital, Shijiazhuang, Hebei, People’s Republic of China.

**Keywords:** anterior cruciate ligament, femoral trochlea, morphology, reconstruction, risk factors, secondary injury

## Abstract

Secondary injury of the anterior cruciate ligament (ACL) is a common concern after anterior cruciate ligament (ACL) reconstruction, and identification of morphological risk factors is essential to prevent these injuries. We hypothesized that abnormal femoral trochlea morphology is associated with secondary ACL injuries after reconstruction. This study aimed to investigate the relationship between femoral trochlear morphology and secondary ACL injuries after reconstruction. A retrospective analysis was conducted on 20 patients who experienced secondary ACL injuries after reconstruction in our hospital between 2017 and 2022 (experimental group), and 40 patients were included in the control group. The following femoral trochlear characteristics were compared between the 2 groups: medial condylar height (MCH), trochlear sulcus height (TSH), lateral condylar height (LCH), trochlear sulcus depth (TSD), trochlear sulcus angle (TSA), medial trochlear inclination (MTI), and lateral trochlear inclination (LTI). The study found that patients in the secondary ACL injury after reconstruction group exhibited the following differences when compared to the control group: decreased MCH (56.33 ± 3.52 vs 59.93 ± 3.24, *P* value = .015), decreased TSD (4.89 ± 1.56 vs 6.98 ± 1.23, *P* value ˂ .001), decreased MTI (12.54 ± 6.57 vs 19.45 ± 6.35, *P* value ˂ .001), and increased TSA (145.23 ± 9.76 vs 139.25 ± 8.42, *P* value ˂ .001). This study demonstrated a significant correlation between abnormal femoral trochlear morphological characteristics and secondary ACL injuries after reconstruction. Decreased MCH, TSD, and MTI along with increased TSA are associated with a higher risk of secondary ACL injury. These data could thus help identify individuals susceptible to secondary ACL injuries after reconstruction.

## 1. Introduction

Anterior cruciate ligament (ACL) injuries are common in young individuals, especially those engaged in sports and physical activities.^[1,2]^ While arthroscopic ACL reconstruction is a widely used treatment for ACL injuries,^[3,4]^ the occurrence of secondary injuries after reconstruction is a significant concern.^[5–7]^ These secondary injuries can have a substantial impact on patients in terms of both physical health and overall wellbeing.^[8,9]^ Investigating the risk factors for secondary injuries after ACL reconstruction is crucial for prevention and management.

Several studies have highlighted the relationship between initial ACL injury and knee joint morphology.^[10,11]^ Factors such as an increased posterior slope of the tibial plateau, femoral intercondylar fossa stenosis, height mismatch of the tibial intercondylar crest, and abnormal femoral trochlear shape have been identified as risk factors for initial ACL injury. Some of these factors, such as the increased posterior slope of the tibial plateau, femoral intercondylar fossa stenosis, and height mismatch of the tibial intercondylar crest, have also been linked to the occurrence of secondary injuries after ACL reconstruction.^[12,13]^

However, the correlation between abnormal shape of the femoral trochlea and secondary injuries after ACL reconstruction remains unclear. We hypothesized that abnormal femoral trochlea morphology is associated with secondary ACL injuries after reconstruction. This study aimed to investigate whether femoral trochlear morphology is associated with the occurrence of secondary injuries after ACL reconstruction. Our research can potentially provide valuable insights into the risk factors associated with secondary injuries, and offer guidance for their prevention and treatment.

This study is important to improve our understanding of secondary injuries after ACL reconstruction and management. This may help clinicians make informed decisions about treatment approaches and strategies for reducing the risk of secondary injuries in individuals who have undergone ACL.

## 2. Methods

### 2.1. Materials and methods

This study was approved by the Institutional Review Board of The Third Hospital of Nanchang (K-ky2023049). We retrospectively included 20 patients with secondary injuries after ACL reconstruction who were treated in our hospital between 2017 and 2022 as the experimental group. Secondary injury after ACL reconstruction was diagnosed using nuclear magnetic resonance imaging and arthroscopy. Exclusion criteria: patients with secondary injury due to ACL reconstruction caused by a car accident or other contact injuries, patients with failure of ACL reconstruction due to postoperative infection, and patients with obvious deviation of the femoral and tibial tunnels during primary ACL reconstruction. Primary ACL reconstruction using a non-autologous ligament (allogeneic or artificial). Patients who underwent primary ACL reconstruction at our hospital between 2017 and 2022 were included in the control group. ACL reconstruction was intact after 2 years of follow-up, and no graft failure occurred. Exclusion criteria: ACL injuries caused by car accidents or other contact injuries. Patients with other ligament injuries (posterior cruciate, medial collateral, and lateral collateral ligament injuries). Patients with contralateral knee joint injuries require normal rehabilitation exercises and activities. According to age (± 2 years), sex, and BMI (± 2), the patients in the control and experimental groups were matched in a 1:2. Finally, 40 patients were included in the control group.

### 2.2. Measurement method

**Figure 1.**
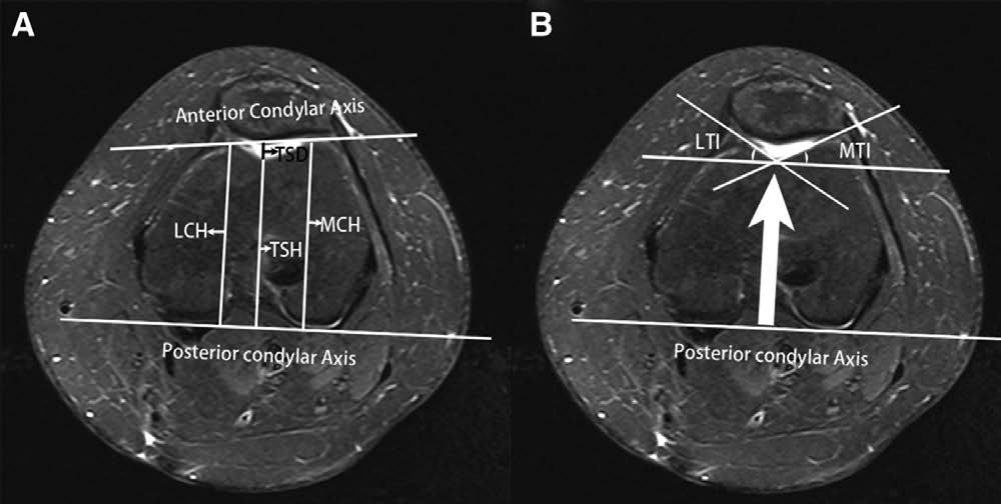
Measurement parameters, including medial condylar height (MCH), lateral condylar height (LCH), trochlear sulcus height (TSH), and trochlear sulcus depth (TSD) (A). Additionally, measurements of lateral trochlear inclination (LTI) and medial trochlear inclination (MTI) are displayed (B).

All patients were scanned using a 3.0-T magnetic resonance imaging scanner (Phillips, Amsterdam, Netherlands) with a 3-dimensional proton density volume isotropic turbo spin echo acquisition sequence using Mimics software (version 17.0; Materialize, Leuven, Belgium) to measure the morphological parameters of the femoral trochlea. Morphological parameters of the femoral trochlea were measured on the largest axial image of the femoral condyle and trochlea.^[14,15]^ First, the posterior condylar axis: the connection between the 2 points of the posterior femoral condyle was determined in the axial direction of the femoral condyle. Medial condylar height (MCH), trochlear sulcus height (TSH), and lateral condylar height (LCH) were determined perpendicular to the posterior condylar line. trochlear sulcus depth (TSD). The medial trochlear inclination (MTI) and lateral trochlear inclination (LTI) were measured, and the trochlear sulcus angle (TSA) was defined as the difference between 180° and the sum of the MTI and LTI (Fig. 1).^[14]^

Two independent observers, with no knowledge of the patient ‘s medical history, measured the aforementioned parameters of the femoral trochlea. Repeated measurements were performed 2 weeks later, and the intra-observer reliability and inter-observer reliability calculated using the intra-class correlation method were 0.85 and 0.93, respectively.

### 2.3. Statistical analysis

Continuous data were presented as mean values and standard deviations. Statistical analysis was performed using the SPSS software (version 18.0, IBM, Chicago, IL, USA). Continuous variables between the 2 groups were assessed using either the 2-independent sample t-test or Mann–Whitney *U* test, selected based on the normality of the data distribution. Statistical significance was set at *P* < .05. A post hoc power analysis was conducted using G*Power 3.1, with the significance level (α) set at 0.05. The statistical power attained was 95.4%. Notably, previous studies aimed at a power of 80%,^[14,16,17]^ underscoring the robustness of the statistical power achieved in this study (95.4%).

## 3. Results

This study included 60 patients, including 20 with secondary injuries following ACL reconstruction and 40 with primary ACL injuries. Among the group with secondary injuries following ACL reconstruction, 12 patients experienced secondary ACL injuries during basketball or football activities, while 8 patients sustained secondary ACL injuries due to sprains while ascending or descending stairs. Notably, all 40 patients in the group with primary ACL injuries had non-contact ACL injuries.

There were no significant differences in age, sex, height, weight, or BMI between the 2 groups (*P* > .05) (Table 1).

**Table 1:** Subject demographics.

	Experimental group	Control group	*P* values
Age, yr	29.9 ± 7.2	28.6 ± 8.2	.228
Sex, male/female	15/5	28/12	.465
Height, cm	177.4 ± 8.2	176.3 ± 9.2	.079
Weight, kg	76.1 ± 4.9	77.3 ± 6.1	.095
BMI, kg/m^2^	22.3 ± 2.8	23.1 ± 2.7	.253

The date of age, height, weight, and BMI were given as the mean and standard deviation. Mann–Whitney *U* test was performed to determine if there was a difference between 2 groups for the age, height, weight, and BMI.

### 3.1. Analysis of risk factors for secondary injury after ACL reconstruction

Logistic regression analysis showed that the MCH in the secondary injury group after ACL reconstruction was significantly lower than that in the control group (56.33 ± 3.52 vs 59.93 ± 3.24, *P* = .015), and TSD (mm) was significantly lower than that in the control group (4.89 ± 1.56 vs 6.98 ± 1.23, *P* < .001). The TSA of the secondary injury group after ACL reconstruction was significantly higher than that of the control group (145.23 ± 9.76 vs 139.25 ± 8.42, *P* < .001), whereas the (MTI was significantly lower than that of the control group (12.54 ± 6.57 vs 19.45 ± 6.35, *P* < .001) (Table 2).

**Table 2:** Comparison of the osseous morphologic measurements among groups.

Variable	Experimental group	Control group	*P* values
Medial condylar height (MCH), mm	56.33 ± 3.52	59.93 ± 3.24	.015*
Trochlear sulcus height (TSH), mm	52.98 ± 3.66	54.19 ± 3.56	.285
Lateral condylar height (LCH), mm	61.89 ± 3.86	63.52 ± 3.21	.153
Trochlear height (TSD), mm	4.89 ± 1.56	6.98 ± 1.23	˂.001*
Trochlear sulcus angle (TSA), °	151.23 ± 9.76	145.25 ± 8.42	˂.001*
Medial trochlear inclination (MTI), °	12.54 ± 6.57	19.45 ± 6.35	.019*
Lateral trochlear inclination (LTI), °	22.36 ± 5.69	22.45 ± 5.21	.142

All date was given as the mean and standard deviation. Mann–Whitney *U* test was performed to detect the significant differences between 2 groups for the TSH and LCH. Two samples t-tests were performed to detect significant differences between the 2 groups for MCH, TSD, TSA, MTI, and LTI.

* Significant difference.

## 4. Discussion

The most important finding of this study is that abnormal femoral trochlea morphology is a risk factor for secondary injury of ACL after reconstruction. This correlation suggests that femoral trochlear morphology could influence secondary ACL injuries. Specifically, the 4 anatomical measurements displayed statistically significant differences. This study demonstrated that decreased MCH, TSD, and MTI along with increased TSA are associated with a higher risk of secondary ACL injury, however, LCH, TSH, and LTI were not.

The anatomical structure of the patellofemoral joint plays a pivotal role in the biomechanics of the knee extension. Van Hafer et al highlighted that different forms of the patellofemoral joint can significantly influence the biomechanical properties of the knee joint.^[18,19]^ Given its critical role in maintaining knee joint stability, the ACL is the ligament most susceptible to injury. Various biomechanical mechanisms can lead to ACL tears, with secondary injuries following ACL reconstruction being of notable concern. In our study, we observed a significant reduction in MCH among patients with secondary ACL injuries. When the medial condyle is smaller, knee flexion during activities, such as squatting or lunging, leads to femoral internal rotation relative to the externally rotated tibia. This, in turn, results in knee valgus and shifts the center of force to the lateral condyle. Consequently, increased ACL tension contributes to the promotion of secondary ACL injuries after reconstruction. This biomechanical principle aligns with previous reports by Liu et al,^[20,21]^ who emphasized that reduction of the medial condyle of the femoral condyle can also predispose individuals to initial ACL injuries.

In our investigation, we extended our analysis to include measurements of the LTI and MTI to substantiate the presence of femoral condylar hypoplasia and gain a deeper understanding of its impact on ACL injuries following reconstruction. Our findings revealed a significant reduction in the MTI among patients with secondary ACL injuries. The medial femoral condyle is crucial for preventing medial patellar dislocation. This reduction in MTI indicates that the femoral trochlea in patients with secondary ACL injury is medially flattened. This alteration may significantly affect the sagittal angle of the patellar tendon and the force transmitted to the knee joint.^[22]^ Because the patellar tendon serves as a connection between the patellofemoral and tibiofemoral joints, the decrease in MTI tends to cause the patella to shift medially as the knee joint extends. Consequently, this leads to an increase in the torque of the tibial internal rotation, thereby promoting the occurrence of ACL injuries following reconstruction.

We observed a significant increase in TSA in the patients with secondary ACL injuries. This finding aligns with previous research on grade-four ACL injuries and highlights the association between elevated TSA and increased risk of a second ACL injury after reconstruction. This relationship is closely related to the influence of the TSA on knee flexion and extension. Carter et al reported that TSA significantly affected patellar orientation in both the frontal and sagittal planes.^[20]^ This in turn affects the magnitude and direction of the force transmitted from the quadriceps femoris to the tibia, thereby increasing the risk of ACL injury after reconstruction. Furthermore, Van Haver et al noted that femoral trochlear dysplasia is rarely an isolated issue and is frequently accompanied by a 10% narrowing of the intercondylar fossa.^[18]^ Numerous scholars have emphasized the role of a narrow intercondylar fossa as a risk factor for secondary ACL injury after reconstruction.^[23–25]^ The significant increase in TSA observed in our study was closely linked to this narrowed intercondylar fossa, potentially exacerbating a second ACL injury after reconstruction. Additionally, our study revealed a significant reduction in the MTI among patients with secondary ACL injuries after reconstruction. This reduction in the MTI may be associated with elevated TSA, further increasing the risk of secondary injuries after ACL injury. Our findings are consistent with those of other colleagues, who conducted a correlation analysis study focusing on the relationship between femoral trochlear dysplasia and ACL injury using CT measurements.^[14,16,26]^ They also reported higher TSA values in the ACL injury group, consistent with our results.

We observed a significant reduction in the TSD in patients with secondary ACL injuries. This finding suggests that femoral trochlear concavity in these patients was notably reduced, causing the patella to shift anteriorly along the sagittal axis. This alteration in patellar positioning may result in a change in the sagittal angle of the patellar tendon, potentially leading to increased forward movement of the tibia relative to the fully extended femur. In simpler terms, a decreased TSD may make knee joint extension easier, which could pose an additional risk of ACL injury in cases with a dominant quadriceps femoris.

The significantly increased TSA observed in our study indicated an elevated risk of secondary ACL injury after reconstruction. This parallels the biomechanical mechanism by which TSA influences the risk of second ACL injury after reconstruction. Pfirrmann et al reported that TSD <5 mm indicates dysplasia, and values <3 mm signify severe dysplasia.^[27]^ In line with this, our findings suggest an increased risk of secondary ACL injury after reconstruction in patients with femoral trochlear dysplasia.

## 5. Conclusion

Our findings highlight a significant association between abnormal femoral trochlear morphology and secondary ACL injuries. Specifically, decreased MCH, reduced TSD, decreased MTI, and increased TSA were significantly associated with secondary ACL injuries following reconstruction. For individuals with these identified risk factors, it is essential to enhance patient education and minimize strenuous activities that can easily lead to secondary ACL injuries during ACL reconstruction. This information can assist clinicians in identifying susceptible individuals and in implementing interventions for the prevention of secondary injuries following ACL reconstruction.

## 6. Limitations

This study had several limitations. First, it was a retrospective case-control study conducted at a single institution, which resulted in a relatively small sample size owing to the strict inclusion criteria. Second, the retrospective design of the study, although valuable, does not replace the challenges and expenses associated with prospective imaging and follow-up of uninjured patients to predict ACL injuries. Third, we focused on the relationship between femoral trochlea morphology and secondary ACL injury, ignoring the relationship between other knee joint morphology and secondary ACL injury. Lastly, the study focus was limited to patients in one region, and its findings may not fully represent the characteristics of secondary injuries following ACL reconstruction in other regions. Future studies should include participants from diverse regions to establish a more comprehensive database.

## Author contributions

**Conceptualization:** Qiangqiang Cai.

**Data curation:** Dongqin Wang.

**Methodology:** Liang Yan, Zhihai Min.

**Project administration:** Xin Wang.

**Software:** Hailin Kuang, Wubing Tang.

**Writing – original draft:** Qiangqiang Cai.

**Writing – review & editing:** Xin Wang.
